# An fMRI study on the neural correlates of social conformity to a sexual minority

**DOI:** 10.1038/s41598-019-40447-3

**Published:** 2019-03-18

**Authors:** M. T. Liuzza, E. Macaluso, P. A. Chiesa, V. Lingiardi, S. M. Aglioti

**Affiliations:** 1 Department of Psychology, Sapienza, University of Rome, Via dei Marsi, 78, 00185 Rome, Italy; 2 IRCCS, Santa Lucia Foundation, Rome, Italy; 3 Department of Surgical and Medical Sciences, “Magna Graecia” University of Catanzaro, Catanzaro, Italy; 4 ImpAct Team, Lyon Neuroscience Research Center (UCBL1, INSERM 1028, CNRS 5292), Lyon, France; 5 Brain & Spine Institute (ICM), INSERM U 1127, CNRS UMR 7225, Boulevard de l’hôpital, F-75013 Paris, France; 6 Department of Dynamic and Clinical Psychology, Sapienza, University of Rome, Via dei Marsi, 78, 00185 Rome, Italy

## Abstract

Social conformity refers to the tendency to align one’s own behaviors, beliefs and values to those of others. Little is known about social influence coming from a minority group. To test whether social pressure from sexual minorities triggers avoidance-motivated behaviors, we explored how being influenced by the preferences of gay peers modifies the behavioral and neural reactivity of individuals defined as in- *vs*. out- groups on the basis of sexual orientation. To this aim, we combined fMRI with a social conformity paradigm in which heterosexual and gay/bisexual (hereafter non-exclusively heterosexual, NEH) individuals provided with male body attractiveness ratings by a fictitious group of gay students may or may not alter their previous rating and may or may not conform to the mean. Behaviorally, conformity to the minority preference was found in in-group NEH more than in out-group heterosexuals. Analysis of BOLD signal showed that social pressure brought about increased brain activity in frontal and parietal regions associated with the detection of social conflict. These results show that members of a sexual majority group display a smaller level of conformity when a sexual minority group exerts social influence. However, the neural correlates of this modulation are yet to be clarified.

## Introduction

Social Conformity refers to the act of changing one’s own thoughts, feelings, and behavior to match the responses of a given group^1^, even when such responses appear blatantly wrong^2^. Generally, social conformity is more pronounced for opinions expressed by in-group than by out-group members^3^. While a number of psychological studies have been conducted on conformity, only recently has this topic attracted the interest of social neuroscience^4^ and the picture emerging from the few fMRI studies on the neural correlates of social conformity is largely incomplete. In a seminal study, Klucharev Hytönen, Rijpkema, Smidts, & Fernández^5^ demonstrated that participants exhibit increased neural activity in rostral cingulate cortex and decreased activity in ventral striatum when providing ratings of facial attractiveness that conflict with those of a group. These two brain regions play a crucial role in conflict monitoring and reward processing, respectively. Also, the social conformity-related choice of changing a decision and accepting an unfair offer was associated with increased activity in the medial prefrontal cortex^6^. Moreover, it has been demonstrated that activity in the dorsomedial prefrontal cortex (dmPFC) is modulated by changes in preference for T-shirts worn by a liked group of peers instead of a disliked group of sex offenders^7^. While it is clear that sex offenders represent a social threat, stigmatization may concern groups that do not pose any objective physical harm, such as gay people. Behavioral and neural data concerning reactivity to the social pressure exerted by this type of group is largely lacking.

To address this issue, we combined fMRI with a modified version of an experimental paradigm that has been successfully used to uncover the neural correlates of social conformity^5^. Our main aim was to investigate whether the natural human tendency to conform to others is contrasted by avoidant behavior towards the stigmatized, minority group. In our study, avoidance was operationally defined not in physical terms, but in terms of willingness to align with aesthetic evaluations expressed by a sexual minority group.

Importantly, conformity in our task was linked to a defining characteristic of the minority group, namely attractiveness towards male bodies. We decided to use an attitudinal object related to sexual orientation in order to maximize the effect under investigation. Indeed, we speculated that homophobic motivations should fuel the willingness to not conform to a sexual minority when the attitude towards an object related to sexual orientation could be perceived by a homophobic participant as a sign of sexual deviance.

In order to assess the extent to which the hypothesized non-conformity behavior could be linked to anti-gay prejudice, we collected explicit measures of modern homophobia (i.e., the modern homophobia scale (MHS)^8^ and right-wing authoritarianism (RWA^9^) that are consistently associated to sexual prejudice towards lesbians and gay men^10,11^. Because these measures are also intimately related to social conformity^12^, we expected them to be negatively related to conformance with gay men ratings of body attractiveness. Moreover, in order to circumvent the effect that social desirability can exert on self-reported attitudes^13–15^, we also used implicit measures of anti-gay attitudes^16^ that assess the strength of the association between positive/negative words and the categories of gay and straight people.

Finally, we expected that some of the brain regions that predict conformist behavior when no in-group *vs*. out-group coding is at play (e.g., the medial prefrontal cortex)^5^ also predict non-conformist behavior when a sexual minority exerts the social influence. This result would support the notion that exposure to out-groups^17^ and sexual minorities^18^ triggers avoidance motivated behaviors.

## Materials and Methods

### Participants

Following recommendations for the number of observations needed to obtain large effect sizes^19^, we tested 32 male participants (mean age = 28.81 years, SD = 7.26). Participants rated their sexual orientation on a Kinsey Scale^20^ ranging from 0 (“exclusively heterosexual”) to 6 (“exclusively homosexual”): fifteen participants rated themselves as exclusively heterosexual, one as equally heterosexual and gay, two as predominantly gay (only occasionally heterosexual), and fourteen as exclusively gay. They were divided into two groups based on these responses: 15 exclusively heterosexual (Kinsey = 0; mean age = 25.53 years, SD = 3.94) and 17 non-exclusively heterosexual participants (NEH, mean Kinsey = 5.71, SD = 0.77, mean age = 31.88 years, SD = 8.27). Although our original plan was to split the sample evenly, we grouped the bisexual participant with the NEH, as studies have found that bisexuals display the same arousal pattern as gay men^21^ and because they are perceived (and stigmatized) as a sexual minority^22^.

All participants had a normal or corrected vision, were free from any contraindication to fMRI and had no history of major psychiatric or neurological problems, as assessed through a questionnaire filled by the participants prior to entering the scanner.

The study was conducted in accordance with the Declaration of Helsinki. The protocol was approved by the independent Ethics Committee of the Santa Lucia Foundation (Scientific Institute for Research Hospitalization and Health Care) on the 17^th^ of May 2013 (Prot. CE/Prog. 403-19). All participants gave written informed consent.

### Visual stimuli

128 virtual male bodies were prepared by using Poser© Pro, a software that creates avatars. We manipulated muscles, thinness, and heaviness of 4 models (Diego, Marcus, Tomo, and Ryan). We then asked an independent group of 16 randomly selected, exclusively heterosexual (Kinsey = 0) female participants (mean age = 27.25, SD = 3.09) to rate the models according to both liking (“how much do you like it?”) and beauty (“how beautiful is it?”) on a 1 (“not at all”) to 7 (“very much”) Likert-type response scale. Because the two measures were highly correlated (*r* = 0.99, *P* < 0.001), we averaged them in a unique attractiveness value. We then excluded 20 images that had mean ratings approaching the minimum possible rating (i.e., the ones judged as the least attractive). Indeed, images that are universally rated very low would systematically prevent our algorithm to generate lower ratings from the fictitious gay group (see below for more details). The final set of 108 stimuli had an average rating of 3.51 (SD = 1.49, range = 1–7). We chose a sample of women under the assumption that they would be more likely to provide more reliable attractiveness rating for male bodies as compared with men, because their self-reported evaluation should be less prone to self-report bias. In fact, even though ratings of male body attractiveness have been provided by males in previous studies^23^, we reasoned that some men who hold negative attitudes towards homosexuality might distort their overt evaluations on the beauty and attractiveness of male bodies. To rule out this possibility we obtained a post-hoc validation of the stimuli by asking fifteen heterosexual men (mean age = 35 years, SD = 5.24) to rate for body attractiveness of the original 128 pictures. The average ratings from the male group closely matched the ones provided by the female group (Spearman’s rho = 0.90, Pearson’s r = 0.90), sharing the 81% of their variance. Also, the two-way intra-class-correlation for the average ratings of the two groups (random raters) was good (ICC2 = 0.75). We also looked at what would have happened had we selected the 20 worst (least attractive) images on the basis of men ratings and found that 15 out of 20 (75%) would have overlapped with the female ones. The Cohens’ kappa on the selected images indicated a substantial agreement (Cohen’s K = 0.70) between the two groups. Finally, we addressed if the ICC2 within each of the groups for the single ratings was indicative of any possible difference in reliability between the two groups. We found that the ICC2 was fair in the female (ICC2 = 0.4), but poor in the male (ICC2 = 0.14) sample.

### Experimental procedure

We employed a modified version of the conformity paradigm used by Klucharev and colleagues^5^. In their fMRI study, the initial judgments of facial attractiveness provided by females were open to the influence of a group of peers (“average European female participant from Milan and Paris”). Participants provided the first rating and attended to the normative group rating in the scanner. The experiments systematically manipulated the ratings of the normative group in order to have a similar amount of normative group pressure towards higher ratings, lower ratings and same ratings. Participants were asked to rate the same female faces again, after the fMRI session to test the effect of the social influence exerted by the normative group.

Here we changed the original procedure in three ways: (1) Participants in Klucharev and colleagues’ study were European (Dutch), the average European rating represented a majority ingroup. In contrast, in our study we wanted to assess the influence of a sexual minority group’s (gay men) on the conformity behavior of minority in-group vs. majority out-group, defined by sexual orientation (i.e. non-exclusively heterosexual vs. heterosexual men); (2) We used male bodies instead of faces with the aim of maximizing the non-conformity effect in homophobic participants. Homophobic individuals may fear assimilation into the prejudiced group when the domain is directly related to sexual orientation^24^. Participants were then asked to rate the attractiveness of the body-stimuli on a 1–9 Likert-type response scale (“how attractive do you find this body?”); (3) We tested the effect of influence immediately after the exposure to the normative group ratings. We decided to show the bodies immediately after the first ratings because bodies are less salient than faces^25^ and therefore harder to be remembered even at an implicit level. Also, each body presented to the participants belonged to one of four identities, which makes even implicit recognition more unlikely and thus may hinder the effect of social influence on the second rating. In contrast, our procedure should maximize the effect. This is important also considering that our design is based on a relatively small sample size. Moreover, because implicit and explicit bias towards gay men is stronger than bias against lesbians^11,16^ participants in our study were all male, while in Klucharev *et al*. they were all female. Our sample might have been thus less susceptible to social conformity than the sample in Klucharev *et al*., as female participants are more likely to conform after undergoing social influence^3^ (but see^26,27^ for a possible alternative explanation of the putative gender differences in social conformity). These considerations motivated us to maximize the conformity effect through a procedure that makes the normative group rating highly salient.

During the fMRI experiment, the participants were asked to rate attractiveness of the male body stimuli and then; (i) in 83% of the trials, were presented with the mean ratings of the same stimuli provided by a putative gay sample (social influence condition); (ii) in 83% of trials were asked to rate the same body stimuli the second time. Crucially, the sample ratings attributed to the gay sample were fictitious and could be higher (Upward social influence), lower (Downward social influence) or the same (Agree) with respect to those provided by the participants. The experimental stimuli had blurred faces in order to encourage participants to focus on flesh properties such as muscularity, a feature that has been shown to be important in determining attractiveness ratings for many gay men^28^. The ratings were given along a Likert-type response scale ranging from 1 (not at all) to 9 (very much) by moving a dark blue circle frame away from the middle value (i.e., “5”) and towards either higher values (right button) or lower values (left button). Participants had three seconds to provide the rating, after which the white circle turned into a bright blue circle frame.

Two to eight seconds later (in 83% of the trials), each participant was shown (through a red circle frame positioned on the scale) the average rating of the same body stimulus given by a ‘gay student sample’ (sexual minority group rating). In reality, this feedback was tailored to each participant by using the following criteria: 33% of trials saw group ratings agree with subject’s ratings, whereas group ratings in the remaining 67% of trials were randomly ±2 or ±3 points above or below the subject’s rating.

When the rating could not be increased/decreased by two or three points, the increase/decrease was shrunk to ±1 or ±2 respectively. This occurred the 18% of trials, with a statistically significant difference between the Het Group (20.5%) and the NEH Group (16.3%, χ^2^(1) = 6.54, *P* = 0.01).

When participants gave extreme ratings (1 or 9), the algorithm automatically gave the same rating (“agreement condition”). Extreme ratings (1 or 9) occurred about 18% of the time. The final distribution of the social influence was the following: 27.4% (NEH = 29.7%, Het = 24.8%) lower ratings, 40.2% same ratings (agreement, NEH = 38.2%, Het = 42.4%) and 32.4% higher ratings (NEH = 32%, Het = 32.8%). The average values of the higher and lower rating were +2.33 and −2.19 respectively. The body stimulus and the mean rating purportedly associated with the minority group (present in the 83% of trials) remained on the screen for three seconds.

**Figure 1:**
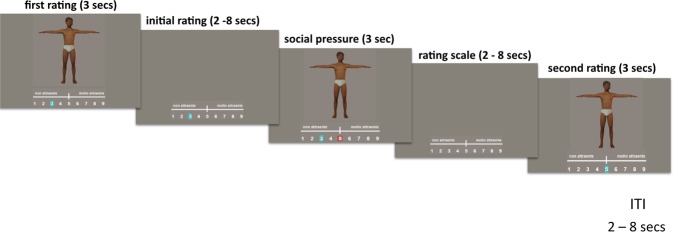
Schematic representation of the experimental task. Structure and timeline of a representative event-trial from the male body attractiveness-rating task. Virtual models were created by the authors (see *Visual Stimuli* for details).

Two to eight seconds after each feedback trial, participants were asked to provide the second rating with the same modality of the first event. Note that by asking for the second rating soon after the fictitious group rating feedback, we hoped to maximize conformist behavior in order to obtain a sensitive measure of how different groups align with the rating of a sexual minority. The choice to have a social influence event in only 83% of trials allowed us to reduce participants’ expectancy and maximize the inter-trial interval variability^29^. At the end of each trial a fixation cross on a grey background was shown for two to eight seconds (see Fig. 1).

### Beauty ratings of non-body stimuli

Before undergoing the fMRI experiment (see below), participants underwent a brief conformity task where they had to provide a first rating on the beauty (“how beautiful do you find it?”) of 12 aboriginal, abstract, expressionist or impressionist paintings^30^ along a 1(“not at all”) to 9 (“extremely”) Likert-type response scale. After providing beauty ratings, participants were shown the putative average rating for the same paintings provided by another group of students who had already participated in the experiment. The social influence remained within ±1 point of the first rating provided by participants. Participants were then shown the same drawing for the second time and asked to provide the second rating. This procedure allowed participants to familiarize themselves with the subsequent male body attractiveness task (i.e., giving a subjective rating and being exposed to social influence before providing the second rating) without disclosing the goal of the study before they had entered the scanner. Note that the paintings never depicted male bodies and the sexual orientation of the normative group was not mentioned.

### Measures of explicit and implicit homophobia

Two measures were used to assess individual differences in the explicit endorsement of conventional social and sexual norms and negative bias towards gays. In particular, to measure explicit anti-gay prejudice, we translated the 22 items of the Modern Homophobia Scale^8^ into Italian, as well as eight Items of the Right Wing Authoritarianism Scale^9^ particularly related to homophobia (e.g., “Homosexuals should be praised for being brave enough to defy “traditional family values” reverse coded) and conventionalism (e.g., “The “old-fashioned ways” and the “old-fashioned values” still provide the best way to live”), an ideological attitude that has been found to be highly predictive of homophobia^31^. For both scales, participants had to rate their degree of agreement on a 6-point Likert-type (1 = “Strongly disagree, 6 = “Strongly agree”) response scale. High scores mean high Homophobia and high Conventionalism respectively.

The two measures were highly correlated (*Spearman rho* = 0.83, *P* < 0.001) to one another. But, since the MHS and the RWA tap into two overlapping but distinct psychological constructs, we decided to narrow our analysis onto the MHS because is the one that is most closely related to homophobia.

Not surprisingly, heterosexuals showed a greater level of explicit (mean MHS = 2.14 ± 0.69 SD) homophobia as compared to NEHs (mean MHS = 1.32 ± 0.44 SD, *T*(30) = 4.01, *P* < 0.001, Cohen’s D = 1.49).

We assessed implicit anti-gay attitudes by asking participants to perform a computerized version of the Sexual orientation implicit association test (IAT). IAT measures the easiness and strength of automatic associations between pairs of social categories (Straight and Gay in the current study) and attributes (good or bad)^14^. We kept the IAT order constant (i.e., “Negative-Gay” and “Positive-Straight” response settings in blocks 3–4 and “Negative-Straight” and “Positive-Gay” response setting in blocks 6–7) across participants to avoid the introduction of additional variance due to the IAT order effect^13^. D scores were computed as suggested by Greenwald *et al*.^13^ and averaged to create a final IAT D score. D values greater than zero reflect an implicit preference for straight relative to gay individuals.

Not surprisingly, heterosexuals showed a greater level of implicit (mean D score = 0.7 ± 0.35) homophobia as compared to NEHs (mean D score = 0.21 ± 0.40, *T*s(30) > 3.71, *P*s < 0.001, Cohen’s D = 1.36).

### fMRI

#### Procedure and apparatus

Participants were positioned in a dimly lit environment while in the scanner. The experimental visual stimuli were presented via a mirror mounted on the MRI head coil (total display size 20° × 15° of visual angle). The visual stimuli were back-projected from a computer monitor (1,024 × 768 screen resolution and 60-Hz refresh rate) onto a screen behind the magnet. Stimulus presentation was controlled with Cogent 2000 (www.vislab.ucl.ac.uk/Cogent/). A fully randomized event-related design was used. Each subject completed three functional runs, and each run consisted of the presentation of 36 stimuli (about 12 per condition) interleaved with a fixation cross (inter-stimulus interval) of jittered duration (1–8 seconds). Each run lasted about 13 min for a total experimental duration of about 40 min.

A Siemens Allegra (Siemens Medical Systems, Erlangen, Germany) operating at 3T and equipped for echo-planar imaging (EPI) acquired functional magnetic resonance (MR) images. A quadrature volume head coil was used for radio-frequency transmission and reception. Head movements were minimized by mild restraint and cushioning. Thirty-two slices of functional MR images were acquired using blood oxygenation level-dependent imaging (3.0 × 3.0 × 2.5-mm thick, 50% distance factor, TR = 2.08 s, TE = 30 ms) covering the entire cortex.

#### fMRI preprocessing

We used the statistical parametric mapping package SPM8 implemented in MATLAB (v 7.1, The MathWorks, Natick, MA) for data preprocessing and statistical analyses. We acquired 1,960 fMRI volumes for each participant, 392 for each of the five functional runs. The first four image volumes of each run were used for stabilizing longitudinal magnetization and were then discarded from the analysis. Preprocessing included rigid-body transformation (realignment) and slice timing to correct for head movement and slice acquisition delay.

Preprocessed data were reviewed for motion using custom software from the Massachusetts Institute of Technology (http://web.mit.edu/swg/software.htm). Functional data were subjected to artifact detection if the derivative of the composite motion exceeded 1.5 mm in any direction. Outlier scans were then modeled in the single-subject General Linear Model by including a single regressor for each outlier scan, with 1 for the outlier and zeros elsewhere. In order to model motion-related noise and spikes in the data, we included the derivatives of the six motion parameters as well as the composite motion parameter in the single-subject GLM^32^. Additionally, we saved the number of outlier scans and the Stimulus Motion Correlation (SMC) for each participant in each condition and checked whether there was a difference between the two groups. To rule out any motion-related group difference we ran a set of unpaired t tests on SCM that showed no statistically significant effect (*T*s(30) < 1.19, *P*s > 0.24). Slice-acquisition delays were corrected using the middle slice as a reference. All images were normalized to the standard SPM8 EPI template, resampled to 2-mm isotropic voxel size, and spatially smoothed using an isotropic Gaussian kernel of 8-mm FWHM. Statistical inference was based on a random effects approach^33^.

### fMRI data analysis

#### First level analyses

The conditions of crucial interest were drawn from the social influence event– of participants being shown the putative mean rating of the fictitious gay group. They were coded as Upward, Downward or Agree conditions depending on whether the mean attractiveness rating from the putative gay sample was higher, lower or identical to the one provided by the participants.

For each participant, a contrast image was estimated from the following comparison: Disagree (mean of Upward and Downward social influence) *vs*. Agree condition. This contrast sought to ascertain any neural modulation induced by the Social influence of the minority (homo or bisexual) on the majority (heterosexual) group.

Furthermore, for each participant’s first rating, data were best fit at each voxel by convolving the hemodynamic response function with the time courses of the following conditions (whose appearance was modeled as an onset with a duration of 0 ms): high rating (7–9), low rating (1–3) and middle rating (4–6). The same modeling was done for the second rating.

All subsequent analyses will focus on the social influence event, as it was brain reactivity to the social influence exerted by a minority group that interested us.

#### Second level analysis

Main effects of Disagreement vs. Agreement: For group random-effect analysis^33^, the single-subjects contrast images for the Disagree *vs*. Agree were entered in a group level analysis performed using an unpaired T-test. The Disagree *vs*. Agree contrast aimed at revealing the main effect of social influence and any interaction with the group.

Statistical maps were initially thresholded at voxel level *P* < 0.001 uncorrected. Results were reported at cluster level *P* < 0.05 corrected for multiple comparisons (Family Wise Error correction, FWE), except when specified otherwise.

Correlations between Disagreement vs. Agreement contrasts and behavioral conformity: In addition to the effects of social influence, we were interested in finding whether any brain activity correlated with conformist/anti-conformist behaviors. To this end, we entered the mean conformity behavior of each participant as a covariate in a one-sample T-test and then tested the slope of the covariate overall. These additional analyses allowed us to test the main effect of the covariate and thus investigate whether, in any brain region, the level of activation for the Disagree vs. Agree contrast co-varied with conformity behavior on a subject-by-subject basis.

Absolute conformist behavior was computed as the mean between the upward conformism (delta of rating after the upward social influence condition - delta of rating after the agreement condition) and the downward conformism (second rating - first rating after the downward social influence condition - delta of rating after the agreement condition) * −1. Downward conformism was multiplied by −1 because, in this case, a negative value is suggestive of conformism.

Statistical maps were initially thresholded at voxel level *P* < 0.001 uncorrected. Results were reported at cluster level *P* < 0.05 corrected for multiple comparisons (Family Wise Correction, FWE), except when specified otherwise.

## Results

### Behavioral results

#### Group differences in attractiveness ratings

Unsurprisingly, NEH gave higher ratings during the first presentation (mean rating = 5.4 ± 0.83 SD) as compared to the Het group (mean rating = 3.2 ± 1.43 SD, t(30) = 5.37, *P* < 0.001, Cohen’s *D* = 1.96). An almost identical pattern of results emerged from the second rating, in which NEH gave higher ratings (mean = 5.45 ± 0.84 SD) as compared to the Het group (mean rating = 3.22 ± 1.44 SD, *T*(30) = 5.45, *P* < 0.001, Cohen’s *D* = 1.99).

#### Conformity Behavior

Previous studies have shown that conformity effects might be due to regression to the mean (RTM)^34,35^, which occurs when a first extreme value is followed by a value that approaches the mean. Thus, the second lower/higher rating might occur not because of upward/downward social influence, but because of RTM. Importantly, our pre-selection led to the exclusion of of extremely unattractive stimuli (as we did not have stimuli judged as too attractive). This procedure was aimed at preventing RTM. Moreover, to further control for possible effects due to RTM, we conducted a linear multilevel model (LMM) on the delta (second rating – first rating) as dependent variable controlling for the first rating^34^.

Unlike traditional statistical methods, LMMs are suitable for analyzing the whole data set while accounting for the non-independence of observations with correlated errors and can easily accommodate unbalanced designs^36^. In this way, we could control for the RTM by adding the first rating as independent variable in our model, as recommended by Yu and Li Chen^34^ and, at the same time, deal with the uneven number of social influence conditions across participants. To this purpose, we used the R package lme4 ver. 1.1–5^37^ and modeled also the random slopes for the first rating and the social influence in order to have a better control of the Type I error, as recommended by Barr and colleagues^38^. Furthermore, we controlled for Age. We then tested for fixed effects using a Type III Wald *χ*^2^ Test through the *Anova* function in *car*^39^. In order to provide a measure of the effect size, we refit the variables using the *standardize* function from the r package *arm*^40^ and report the *β* coefficients.

The following formula describes the model that was tested using the Wilkinson notation (ID stands for the effect of the participant):

Delta ~ (1 | ID) + Age + Social influence + Group + First Rating + (0 + Social influence | ID) + (0 + First Rating | ID) + First Rating: Group.

**Figure 2:**
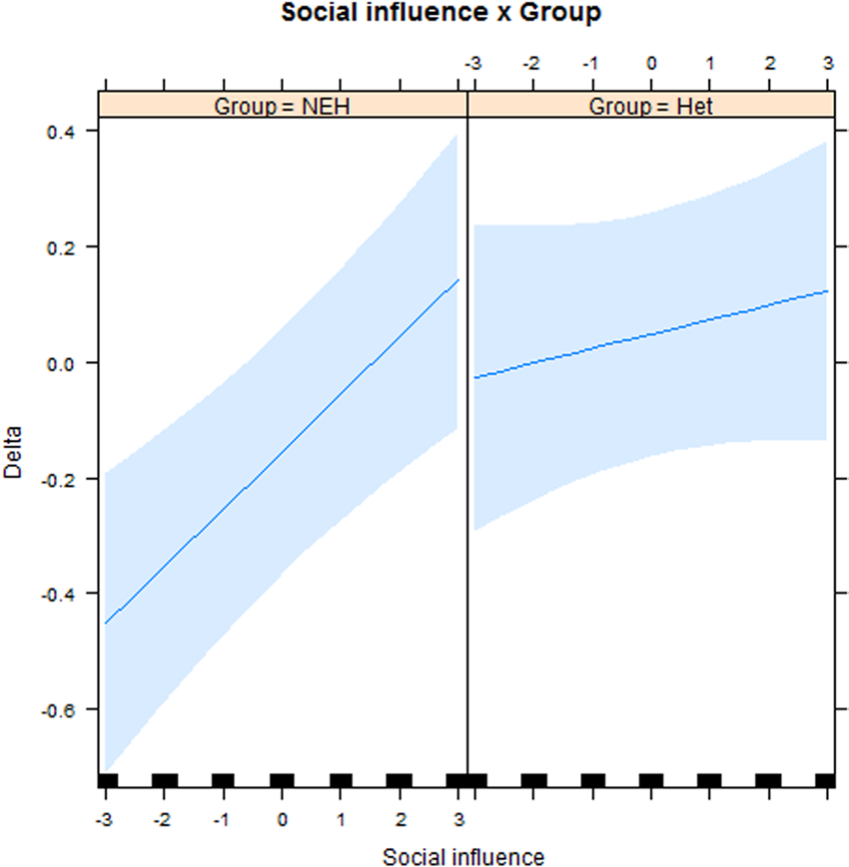
Behavioral results. The plot represents the interaction between Social influence (from −3 to +3) and Group (Het = Heterosexuals, NEH = Non-exclusively heterosexuals) in determining the difference between the first and the second attractiveness rating (Delta). Light blue shades represent 95% confidence bands, rugs represent the number of observations for each value.

Our results showed a main effect of the first rating (*β* = −*0.17, χ*^2^(1) = 6.73, *P* = 0.009), social influence (*β* = *0.17, χ*^2^(1) = 15.87, *P* < 0.001) of the Group (*β* = −*0.18, χ*^2^(1) = 3.87, *P* = 0.049), while the effect of Age was not statistically significant (*β* = −*0.001, χ*^2^(1) = 0.73, *P* = 0.39). Importantly, we found also an interaction between social influence and Group (*β* = −*0.20*, *χ*^2^(1) = 4.11, *P* = 0.04, see Fig. 2).

As Fig. 2 shows, the effect exerted by Social Influence on conformity behavior was remarkably larger in the NEH group as compared to the Het group. Nevertheless, a marginally significant effect of social influence was found also in the Het Group (*β* = *0.02*, *χ*^2^(1) = 3.78, *P* = 0.052).

Overall, this pattern of results showed that, even though both groups are influenced by the fictitious gay group rating to some extent, the NEH group was significantly more influenced.

We tested whether the difference in explicit prejudice could be related to the level of conformity. We found that MHS is not predictive of conformity behavior, when taking the regression towards the mean into account, as the interaction between MHS and Pressure did not reach statistical significance (*β* = *0.09*, *χ*^2^(1) = 0.57, *P* = 0.45). We tested the same hypothesis for the IAT, and the interaction between the IAT D scores and Pressure did not reach statistical significance (*β* = −*0.06*, *χ*^2^(1) = 0.24, *P* = 0.62).

To rule out the possibility that the two groups differed in their tendency to conformity in a non- specific way, we analyzed the data from the painting beauty ratings task (i.e., the familiarization task) of 30 participants (we lost the data of two participants, one from each group) by means of an LMM model with only the random intercept, given the small number of observations per participant. While we did not find any statistically reliable main effect of conformity (*Χ*^2^(1) = 2.89, *P* = 0.089), note that there is a trend in the expected direction. Thus, although the available trials were limited in number, a positive relationship between social influence and rating change (*β* = 0.22) was observed. Importantly, the interaction between group and influence was far from being statistically significant (*β* = 0.06, *Χ*^2^(1) = 0.06, *P* = 0.8). Thus, any tendency towards social conformity in the beauty ratings of non-male-body related paintings did not differ between the two groups.

In order to provide evidence in support of the null hypothesis (the lack of interaction between group and conformity), we also analyzed the data using *BayesFactor* R package^41^. We compared the model with the interaction between group and social influence to the model with only the group and social influence main effect. We found that the model with only the main effects has a Bayes Factor of 32553 (a decisive evidence against the interaction, according to^42^) compared to the interaction model.

To sum up, the analyses of the ratings given in the painting beauty task (in which no information on the sexual orientation of the fictitious group was provided) showed that the heterosexual group and the NEH group do not differ in their general tendency to conform to others.

### fMRI Results

#### Effect of social influence

**Figure 3:**
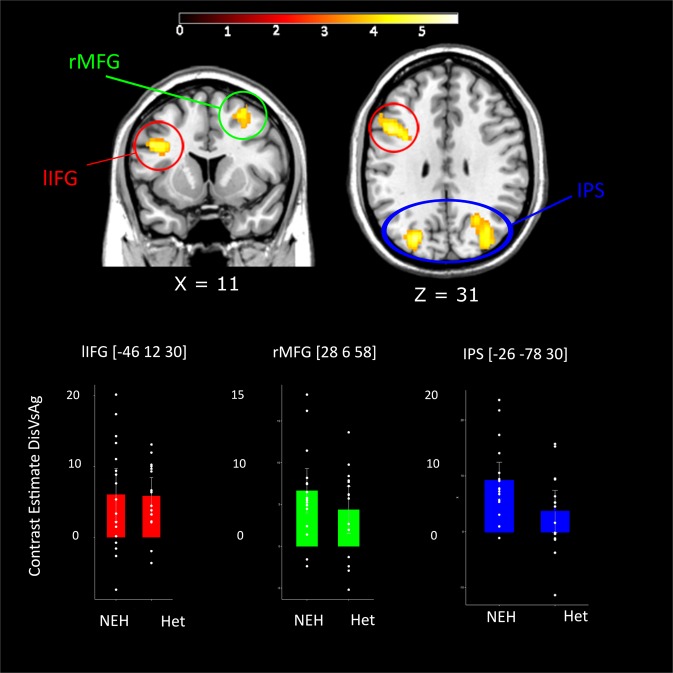
Whole brain Social Influence effects (Disagreement *vs*. Agreement). Whole brain activation for the Disagree *vs*. Agree contrast displayed in the axial and coronal section. The bar plots at the bottom show the means and the 95% Confidence Intervals for the contrast estimates for the Disagree – Agree contrast in each group. The bar graphs are overlaid with the single data points.

We first investigated the hemodynamic responses related to exposure to the Social influence (i.e., Disagree *vs*. Agree) of the sexual minority group. The results of these analyses showed the activation of a fronto-parietal network, including the right middle frontal gyrus (rMFG), the intra parietal sulcus (IPS) bilaterally, and the left inferior frontal gyrus (lIFG). See Table 1 and Fig. 3.

**Table 1:** Significant brain activations in different social influence conditions (*vs* agreement).

Brain regions	Disagree *vs* Agree				
	No. of voxels	x	y	z	Z
Right Intra Parietal Sulcus	1690	42	−40	40	4.41
Right Superior Parietal Lobule/Precuneus		20	−64	52	4.31
Right Middle occipital Gyrus		34	−72	30	3.88
Left Intra parietal Sulcus	1214	−28	−48	42	4.37
Left intraparietal Sulcus		−26	−78	30	4.33
Inferior Parietal Lobule		−30	−60	44	3.99
Left Inferior Frontal Gyrus/Pars Triangularis	499	−46	12	30	4.30
Left Precentral Gyrus		−38	4	36	4.13
Right Middle Frontal Gyrus	339	28	6	58	4.66

Coordinates of local maxima (x, y, z) are defined in Montreal Neurologic Institute (MNI) stereotactic space. Z values referred to Disagree vs. agree contrast. *P* < 0.05 (FWE, cluster level) or *P* < 0.05 (FWE, voxel level). Number of voxels in each cluster are reported only in correspondence to the local maximum of each cluster and omitted in the following maxima.

We did not find any significant cluster for the interaction Group x Social influence effect.

#### Correlations between Disagreement vs. agreement contrasts and behavioral conformity

We found a negative correlation between the Disagree *vs*. Agree contrast estimate (CE) and conformity behavior across all participants in three voxels (p < 0.05, FWE corrected) within a cluster that includes middle superior frontal gyrus/dorsomedial prefrontal cortex (dmPFC).

However, a closer inspection of the scatterplot displaying the relationship between the CE and the Conformity behavior in these voxels revealed the presence of an outlier participant (NEH) whose CE deviated more than three standard deviations from the rest of the sample. When that participant was dropped from the analyses, no significant cluster and/or voxel was found significant when correcting for the FWE.

## Discussion

Social conformity is a robust, evolutionarily rooted phenomenon likely motivated by the need for a given individual to be approved by other individuals and to avoid conflicts with them. The phenomenon may be particularly patent when those who conform and those who lead others to conform belong to the same social group^3^. Pioneering studies on the neural underpinnings of social conformity highlight the important role of reward-related (ventral-striatal) and conflict-related (medio-frontal) regions in mediating the adjustment to the opinions of others^43,44^. Moreover, activity in medio-frontal regions tracks the direction of preference change from one’s own opinion towards or away from that of liked *vs*. disliked groups^7^. However, to the best of our knowledge, no study has thus far explored the neural systems activated when social conformity is induced by a group defined as in- or out-group on the basis of sexual orientation. To fill this gap, we tested whether a minority group of sexually stigmatized gay men would instigate social conformity to a different extent in a minority group of peers (i.e., exclusively gay or bisexual individuals) as compared to a majority group of exclusively heterosexual individuals. Moreover, we explored how any modulation of social conformity may be reflected in the activity of brain areas involved in social influence. Importantly, the perceptual, cognitive and emotional dimensions tapped by our conformity task involved a feature conspicuously associated with the group that should lead to conformity, namely the physical attraction towards male bodies that gay men may typically experience.

One important behavioral result of our study is that social conformity, as indexed by the alignment of one’s own ratings of male body attractiveness with the ratings given by a sexual minority of gay students, was stronger in gay and bi-sexual as compared to heterosexual people. More specifically, alignment in NEH ratings (as indexed by the delta between the first and second rating in the direction of the social influence) significantly differs from that of the heterosexual participants. Tellingly, no differential conformity was instigated by a fictitious group whose sexual preference was not specified. Indeed, no differences in the delta between first and second rating in the direction of the social influence was found in the control task where participants had to rate the attractiveness of paintings that did not depict male bodies. This finding rules out any group difference in the general tendency to conform to others and suggests that the observed difference in conformity may be determined by the sexual orientation of the influencing group.

With that being said, we acknowledge that the aesthetic rating of a sexually neutral stimulus may constitute a suboptimal control for our main task. Future studies should focus more systematically on the evaluation of sexually neutral stimuli along with the manipulation of the group that exerts the social influence (majority vs. minority).

It has been argued that results from studies on conformity may be undermined by the phenomenon known as regression to the mean (RTM)^34,45^, that occurs when an extreme measurement at time 1 is more likely to be followed by a less extreme value at time 2. This makes a natural variation in repeated measures to look like an experimentally-induced change^46^.

In our study, we controlled for the possible confounding effect of RTM by entering the first rating as a covariate of no interest, as recommended by^34^ (although it has been held that this method may even underestimate the effect of social influence^35^).

Seminal findings from Moscovici and colleagues have shown that minorities may exert an influence on the majority^47^ (see^48,49^ for a recent review). Importantly, however, this form of influence is most effective when informational social influence is at play^50^. Informational influence occurs when people rely upon others’ behaviors and beliefs in order to be accurate^1^. Crucially, however, our participants performed subjective evaluation of attractiveness, rather than accurate estimations of objective property of the stimuli (e.g. specific body features). In cases of so-called normative social influence, where opinion tasks are at stake, social identity^51^ seems to be fundamentally important^48^. This is consistent with our findings, where the conformity behavior is stronger among participants – NEH - who can identify with the normative group – the fictive gay group.

Unfortunately, the lack of a condition where a sexual majority group exerts the social influence prevented us from testing whether the social influence from out-group causes a decrease in the conformity behavior as compared to the ingroup in each group, thus limiting the scope of our findings.

It might be argued that our decision to split the sample into groups (NEH and Het) might be somewhat arbitrary, since the NEH includes individuals who placed themselves in intermediate positions along the Kinsey scale. Our choice had a two-fold motivation. Firstly, the small number of participants who did not consider themselves as exclusively gay or exclusively heterosexual is too small to allow any reliable estimate (N = 3, one bisexual and two predominantly gay - only occasionally heterosexual). Secondly, from a social identity standpoint, bisexuals are definitively perceived – and likely to perceive themselves – as members of a sexual minority^22^.

Another potential weakness is that we had selected our best 108 stimuli on an original set of 128 on the basis of the ratings of a sample of heterosexual women but then run the study on a sample of heterosexual and NEH men. Importantly, however, a post-hoc validation of our stimuli on a sample of heterosexual men demonstrated a large overlap in terms of shared variance, agreement on the stimuli selection and in terms of agreement on the ratings. Furthermore, we found that the reliability of the ratings within the female group was fair, whereas the reliability of the ratings within the male group was poor, a result that further justifies our prior selection of the stimuli. It is also important to emphasize that this selection procedure was aimed at alleviating floor effects on the ratings that could have biased the results because of the RTM phenomenon^34,35,45,46^.

Our behavioral results are in line with the findings summarized in the meta-analysis from Bond and Smith^3^ who found that when the influencing group is perceived as an out-group, social conformity decreases, although this effect was not statistically significant when controlling for other relevant factors (e.g. culture, gender, date of the study). Moreover, a recent study found that, similarly to what found in our study, conformity was attenuated when influence was exerted by an out-group (e.g., “Trump supporters” when participants were Trump opponents)^52^. Conformity was even reversed when participants perceived the out-group as more threatening. In our study we did not find evidence for a blatant reversal of conformism, but rather an attenuation of conformism when influence came from an out-group sexual minority. This might be due to the fact that very few of the participants perceived NEH as a moral-threatening group, considering that our sample was recruited mostly from Psychology college students who typically hold liberal views^11^. Indeed, although the Heterosexual group showed higher levels of explicit homophobia, their average score on the MHS was 2.39 on a scale whose final scores could range from 1 to 6.

It is worth noting that neither explicit nor implicit homophobia was significantly related to conformity, despite the two groups significantly differed on both dimensions. This might suggest that perhaps, more than prejudice *per se*, it is the relevance of the opinion of an in-group that matters, especially when the behavior under scrutiny (the evaluation of male body attractiveness) is relevant to the group categorization. In other words, it might be that gay men conformed more to the opinion of their ingroup peers because the required rating concerned a feature highly relevant to people who are more likely to find a male body as attractive. In contrast to previous studies where the in-group *vs*. outgroup source of influence was experimentally manipulated^53,54^, the quasi-experimental nature of the current study prevents us from drawing any strong causal conclusion.

It is well known that sexual minorities suffer more distress than heterosexual people (e.g.^55,56^), and conformity behavior may be seen as a way to cope with social exclusion^57^. Although social exclusion may mediate the stronger conformity behavior with the ingroup in the social minority group, we did not collect any measure that could corroborate this hypothesis. In any case, the small sample size would have prevented us to test for any mediation. Future research is warranted to test the hypothesis that the effect of perceived social exclusion on conformity behavior among minorities may mediate the stronger conformity behavior with the minority ingroup found in our study.

It could be argued that choosing an attitudinal object that is related to sexual orientation (i.e., male bodies attractiveness ratings) may conflate the effect on conformity with the fear of being labeled as gay that some male participants might experience. From this perspective, it would have been more straightforward to design a study in which a sexual orientation unrelated object was used, for instance the abstract paintings used in the familiarization task. However, we submit that our choice has strengthened, rather than conflated, the effect under investigation. In fact, had the primal motivation of the participants been to not appear attracted to male bodies, then the best strategy would have been to just provide low attractiveness ratings in the first place, which is not what we actually observed.

The analyses of BOLD signal revealed a main effect of social influence in a fronto-parietal network that included the intraparietal sulcus, bilaterally, the left inferior frontal and the right middle frontal gyrus, a set of areas that were also activated in previous studies on social conformity (e.g.^5,7^). Interestingly, the dynamic involvement of fronto-parietal regions in the condition of matching *vs*. mismatching between individual and group opinion has been demonstrated also using magnetoencephalography^58^. Our results further support the electrophysiological evidence in favor of a posterior source^58^ of the feedback-related negativity (FRN), an event-related brain potential (ERP) component associated with social influence^59,60^.

We failed to find any statistically significant result in the BOLD signal pointing at a group difference in the way the brain responds to disagreement with an in-group (*vs*. out-group). Although visual inspection of the different contrast estimates for the two groups seems to suggest a bigger response in the NEH group, thus suggesting a greater sensitivity of this group to the social influence of their ingroup members, these differences do not pass the statistical threshold after FWE correction at the whole brain level. This might be due to either noise or to the paucity of our sample size (N = 16 per group) and/or to a negligible effect size. Future studies that address more directly these issues are needed.

The whole brain analysis performed in the present study on the relationship between neural reactivity to social influence and conformist behavior indicates that activity in voxels belonging to the dorsomedial prefrontal cortex (dmPFC) seems to predict non-conformist behavior. The involvement of the dorsomedial prefrontal cortex (dmPFC) may be relevant for making decisions based on stereotypical beliefs^61^ and for stereotyping processes at large^62^. In fact, Saxe and Wexler^63^ showed that dmPFC activity increases when a person is described as a foreigner, possibly because it is hard to build up expectations about unfamiliar people. Importantly, the dmPFC has been shown to be preferentially activated in social influence tasks where the participants either disagreed with a liked group of Caltech students or agreed with a disliked group of sex offenders^7^. It is worth noting, however, that a further inspection of our data suggested that this effect could be driven by a single outlier observation and therefore the interpretation of our results should be taken cautiously.

In conclusion, our study provides novel knowledge about how intergroup dynamics in the sexual orientation domain can significantly affect the strength of conformity behavior. Thus, it appears that humans are less prone to the influence of out-groups who belong to a sexual minority. At the neural level, although dorsomedial prefrontal cortex (dmPFC) seems to relate to non-conformist behavior, we failed to find any compelling evidence for the link between neural activity and conformity driven by sexual orientation.

## Data Availability

The datasets generated during and/or analyzed during the current study are available from the corresponding author on request. Since the data set contains sensitive information on participants sexual orientation we cannot make the dataset public.
